# ﻿Description of a new species of *Stauropathes* (Anthozoa, Antipatharia, Schizopathidae) from Puerto Rico

**DOI:** 10.3897/zookeys.1231.136967

**Published:** 2025-03-13

**Authors:** Jeremy Horowitz, Mina Barajas, Luke J. McCartin, Samuel A. Vohsen, Santiago Herrera

**Affiliations:** 1 Department of Invertebrate Zoology, Smithsonian Institution, National Museum of Natural History, Washington, DC, USA; 2 Department of Zoology, California State Polytechnic University, Humboldt, Arcata, CA, USA; 3 Department of Biological Sciences, Lehigh University, Bethlehem, PA, USA; 4 Lehigh Oceans Research Center, Lehigh University, Bethlehem, PA, USA

**Keywords:** Black coral, genome skimming, molecular phylogenetics, morphology, targeted capture, taxonomy, ultra-conserved elements

## Abstract

A new species of black coral, *Stauropathesmonopinnata***sp. nov.**, represented by two specimens collected 738 m and 1604 m deep off Puerto Rico and Hawaii, respectively, is recognized in the family Schizopathidae. The new species is characterized by a monopodial, unbranched corallum; simple, suboppositely arranged pinnules in two anterolateral rows along the stem with nearly 90° distal angles, spaced 12–17 mm apart in a row, and with smooth and triangular spines 0.05–0.08 mm tall; and polyps 4–9 mm in transverse diameter. A phylogeny composed of 90 taxa representing species in Schizopathidae and Cladopathidae (rooted in Cladopathidae) was reconstructed from 794 nuclear loci to show their systematic relationships. Herein, we provide morphological and molecular evidence to show that this new species is distinct from other species in the genus *Stauropathes*.

## ﻿Introduction

Black corals are a group of hexacorals that occur in all oceans (except northern Arctic Ocean and hydrothermal vents) and depths from just below the surface to 8600 m. The taxonomy of black corals is undergoing an order-wide taxonomic review with novel data like next-generation sequencing techniques, which are providing greater phylogenetic resolution than single locus or even full mitochondrial genomes (Horowitz et al. 2020; Quattrini et al. 2024).

During the Schmidt Ocean Institute expedition FKt230417, titled ‘Health Diagnostics of Deep-Sea Coral’, 74 black corals were collected from the deep waters off Puerto Rico. This collection recently led to the discovery of new species representing a novel genus and family (Horowitz et al. 2024). Upon further examination of this collection, we discovered a monopodial, unbranched colony characterized by simple suboppositely positioned pinnules, which is a common feature among species in *Bathypathes* Brook, 1889. However, it differed from *Bathypathes* species in that the distances between the members of each subopposite pair of pinnules were extremely small, almost opposite, the colony had wide spaces between pinnules in a row, and the longest pinnules were also the lowermost ones on the stem, which are common among species of *Stauropathes* Opresko, 2002. A second specimen, collected from deep waters off Hawaii, during the National Oceanic and Atmospheric Administration expedition titled: ‘Hohonu Moana: Exploring Deep Waters off Hawai’i’ possesses these same morphological characteristics. Both specimens, containing *Stauropathes*-like characters with the exception that they were not branched, were sequenced using genome skimming. We bioinformatically extracted conserved element loci to build a phylogenetic tree and place the new species within the Schizopathidae Brook, 1889. This analysis revealed that these specimens are more closely related to each other than to any other currently sequenced species, both falling as sister to nominal *Stauropathes* species including the type specimen of the type species, *Stauropathesstaurocrada* Opresko, 2002 (USNM 98846). Herein, we present morphological and molecular evidence to describe this new-to-science species.

## ﻿Material and methods

### ﻿Specimen collection and deposition

**Figure 1.**
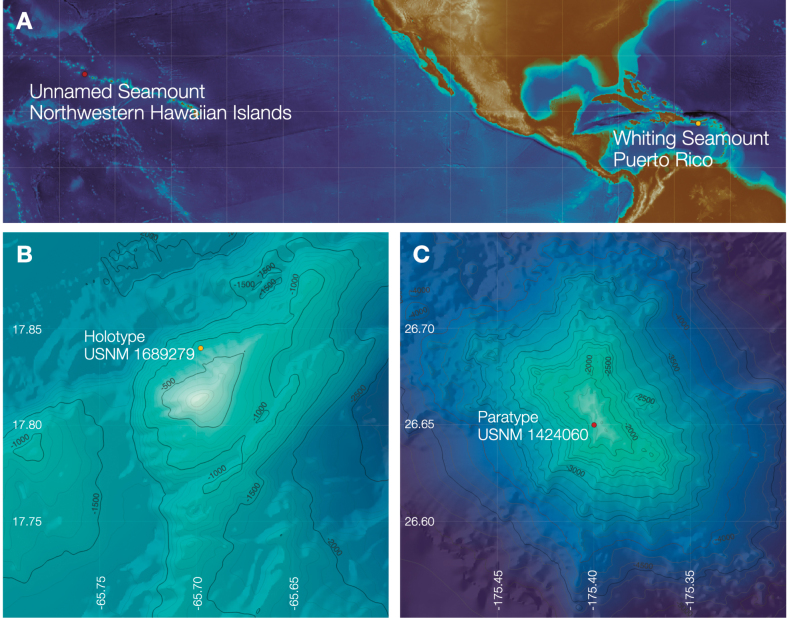
Locations where the holotype (yellow dot) and paratype (red dot) were collected.

The holotype was collected at Whiting Bank, 25 km southeast of Puerto Rico, 738 m deep during the Schmidt Ocean Institute expedition FKt230417 entitled: ‘Health diagnostics of deep-sea coral’ (Fig. 1) onboard the R/V *Falkor (too)* in 2023. The complete colony was collected using a manipulator arm of the ROV *SuBastian*. The paratype was collected 250 km southeast of Midway Atoll, Hawaii at a depth of 1604 m during the National Oceanic and Atmospheric Administration expedition entitled: ‘Hohonu Moana: Exploring Deep Waters off Hawai’i’ onboard the R/V *Okeanos Explorer* in 2016. Both specimens are deposited in the collections of the National Museum of Natural History (NMNH), Smithsonian Institution, Washington DC.

### ﻿Morphological analyses

The morphological characters of the specimens representing the new species were compared with nominal and currently accepted species in the genera *Bathypathes* and *Stauropathes*.

The skeletal spines were examined by cutting small fragments of the skeleton and removing the tissue with bleach and a sonicator. The fragments were then mounted on stubs and coated with a 30–40 nm thick layer of 60% gold: 40% palladium and imaged using a Zeiss EVO MA 15 scanning electron microscope (SEM). SEM stubs are deposited at the NMNH. SEM stub numbers are from a series established by the authors at the NMNH. Spine height was measured as the distance from the spine tip to the middle of the base of the spine. Polyp in transverse diameter was measured as the distance from the distal edge of the distal lateral tentacles to the proximal edge of the proximal lateral tentacles. Branch diameter was measured near the base of the branch. Distance between pinnules of the same pair was measured from the middle of the base of the lower pinnule to the middle of the base of the upper pinnule.

**Figure 2.**
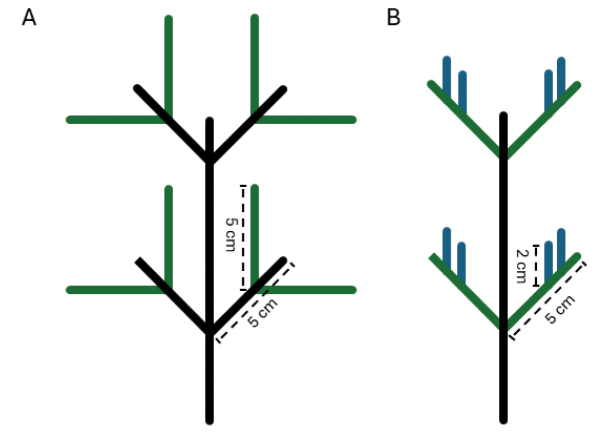
**A** example of a branching colony with a stem and pinnulated branches (black lines) and primary pinnules (green lines) **B** example of an unbranched colony with a stem (black line), primary pinnules (green lines), and secondary pinnules (blue lines).

We define the distinction between pinnule and pinnulated branch based on the similarity or dissimilarity of branching patterns at successive branch or ramification orders. If a ramification exhibits the same pinnulation pattern and length as its immediate lower-order ramification, it is a pinnulated branch, and its pinnules are first-order pinnules (Fig. 2A), as seen in *Telopathes* MacIsaac & Best, 2013 and *Stauropathes*. Conversely, if the pinnulation pattern changes at higher orders, each ramification represents a distinct pinnule order (Fig. 2B), as in Myriopathes Opresko, 2001.

### ﻿Molecular analyses

DNA was extracted from the holotype specimen USNM 1689279 alongside all specimens collected in Puerto Rico using the AutoGen GenePrep (AutoGen, Holliston, MA, USA) in the Laboratories of Analytical Biology (LAB) at the NMNH. GenePrep automatically extracts DNA using Phenol-Chloroform purification with high throughput. Extracted DNA was quantified using the QuantIT 1X dsDNA high-sensitivity kit (ThermoFisher Scientific, Waltham, MA, USA). Sequencing libraries were prepared in half reactions using the New England Biolabs (Ipswich, MA, USA) Next Ultra II FS DNA Library Prep kit with incubation times of either 7.5 or 8.5 mins for enzymatic shearing. Bead cleanups were performed using KAPA pure beads (Roche Diagnostics, Indianapolis, IN, USA) and an Opentrons robot with a bead ratio of 0.8X (Long Island City, NY, USA). Using Y-yoke adapter sequences, samples were indexed with unique combinations of iTru i5 and i7 barcode indices before pooling (Glenn et al. 2019). Library preparation for some samples failed, and libraries for these samples were re-prepared after further purifying the DNA extract using a Qiagen PowerClean Cleanup Pro kit following the manufacturer’s instructions (Qiagen, Hilden, Germany).

For the paratype specimen from Hawaii (USNM 1424060) and all other specimens that were collected outside Puerto Rico waters, DNA was extracted with the DNeasy Blood and Tissue Kit (Qiagen, Germany), cleaned with the Qiagen Power Clean Pro kit, and concentrations were estimated using a High Sensitivity Qubit 4 Fluorometer (Invitrogen, US). High molecular weight DNA was sheared using a QSonica Inc Sonicator Q800R to a target size range of 400–800 bp and then checked via gel electrophoresis on a 1.5% agarose gel. Afterwards, DNA libraries were prepared with the Kappa Hyper Prep protocol using a ½ reaction with iTruSeq adapters and dual indexes (Glenn et al. 2019) following Quattrini et al. (2018).

For all specimens included in this study, DNA extraction and library preparation were conducted at LAB. Paired-end sequencing (150 bp) was performed on an Illumina - NovaSeq X Plus at the Oklahoma Medical Research Foundation Genomics Facility with other samples to obtain 10M paired-end (PE) reads (150 bp) per sample. Raw reads are deposited in the short read archive (SRA) of the National Center for Biotechnology Information (https://www.ncbi.nlm.nih.gov/).

### ﻿Phylogenetic analysis

The conserved loci were obtained from the high-throughput sequencing data. Raw reads were trimmed using Trimmomatic v. 0.35 (Bolger et al. 2014) and assembled using Spades v. 3.15 (Prjibelski et al. 2020). Ultra-conserved elements (UCEs) and exon loci were extracted from the assembled data using the hexacoral-v2-baitset (Cowman et al. 2020), following the Phyluce pipeline (https://phyluce.readthedocs.io/en/latest/tutorials/tutorial-1.html) (Faircloth 2016) with modifications: minimum-identity and minimum-coverage thresholds set to 70%. These data were combined with loci that were determined to be conserved across black corals previously (see Horowitz et al. 2020, 2022, 2023a, 2023b, 2024). Loci were internally trimmed and aligned with MAFFT v. 7.130 (Katoh and Standley 2013). Then, phyluce_align_get_only_loci_with_min_taxa was used to obtain all loci with 50% taxon-occupancy, which were concatenated using phyluce_align_concatenate_alignments. 50% taxon-occupancy was chosen because it maximizes the number of loci incorporated into the phylogenetic reconstruction and, based on previous phylogenetic studies, yields high support values (Horowitz et al. 2023a, 2024). The phylogenomic inference was conducted on the concatenated dataset using maximum likelihood analysis in IQ-TREE v. 2.1 (Minh et al. 2020). A partitioned analysis (Chernomor et al. 2016) was conducted on the dataset using the best model for each locus [-m TESTMERGE (Kalyaanamoorthy et al. 2017)]. Ultrafast bootstrapping [-bb 1000 (Hoang et al. 2018)] and the Sh-like approximate likelihood ratio test [-alrt 1000 (Anisimova et al. 2011)] were also selected. All analyses were run on the Smithsonian’s High-Performance Computing Cluster (https://doi.org/10.25572/SIHPC) housed at the Herndon Data Center, in Herndon VA, except for the phylogeny, which was built using FigTree v. 1.4.4 and R v. 4.4.1.

## ﻿Taxonomic results

### ﻿Family Schizopathidae Brook, 1889

#### ﻿Genus Stauropathes

Taxon classificationAnimaliaAntipathariaSchizopathidae

Opresko, 2002

33DEB4EA-C7CF-508B-913D-C18E490C9BA3

##### Diagnosis (emended from Opresko 2002).

Corallum monopodial, unbranched or branched and pinnulate to the first order. Pinnules in two lateral or anterolateral rows and arranged in subopposite pairs. Spines smooth, triangular, and laterally compressed. Polyps 3–9 mm in transverse diameter.

##### Type species.

*Stauropathesstaurocrada* Opresko, 2002 (by original designation).

##### Type locality.

North-central Pacific Ocean.

##### Remarks.

This study demonstrates that the unbranched corallum with bilateral and subopposite pinnules is polyphyletic, occurring in the genera *Bathypathes* and *Stauropathes*, warranting emendation to the generic diagnosis for *Stauropathes*. Further, the two genera do not form monophyletic groups, where *Stauropathes* spp. (CMNI 2023-0258, USNM 1404493, and USNM 1424220) fall into the clade that consists of a majority of *Bathypathes*, while *Bathypathesalaskensis* Opresko & Molodtsova, 2021 (USNM 1013749) falls into the clade that consists of a majority of *Stauropathes*. Another finding of this study is that *Telopathes* is not polyphyletic, contradicting Cruz et al. (2024); however, additional sequence data from these three genera may suggest more complicated relationships, possibly fueled by hybridization and/or introgression, which has yet to be formally tested for any species in the order.

#### ﻿ Stauropathes monopinnata

Taxon classificationAnimaliaAntipathariaSchizopathidae

Horowitz & Barajas sp. nov.

6ADC2AFA-B9DC-5DCD-B5B1-CEF1B61F1C4E

https://zoobank.org/86C16844-9D66-42CF-9337-CF9CED4A8375

**Figure 3.**
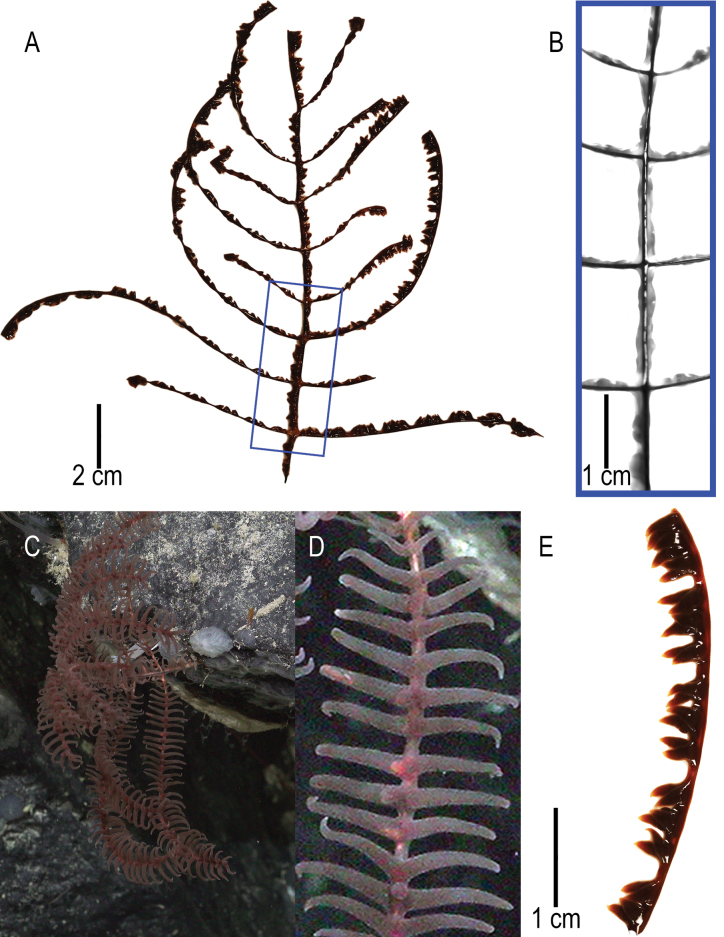
*Stauropathesmonopinnata* sp. nov. holotype: USNM 1689279 **A** collected colony with **B** blue rectangle showing zoomed-in view of pinnule pattern **C** in situ image of colony **D** in situ image of colony showing zoomed-in view of polyp characteristics **E** section of pinnule showing polyp characteristics.

**Figure 4.**
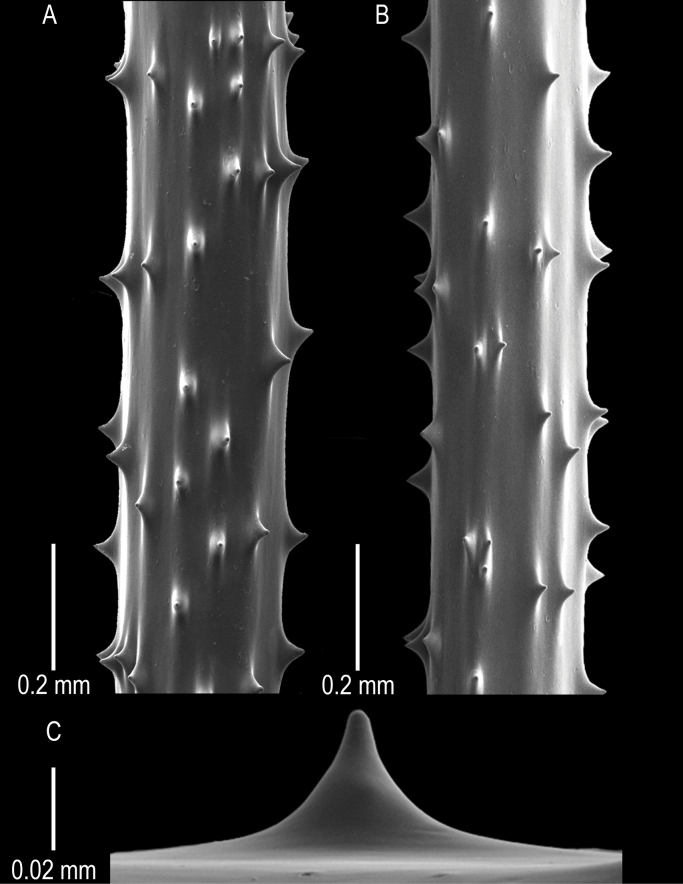
*Stauropathesmonopinnata* sp. nov. holotype: USNM 1689279 **A, B** sections of pinnules showing skeletal spines **C** zoomed-in view of a singular skeletal spine.

**Figure 5.**
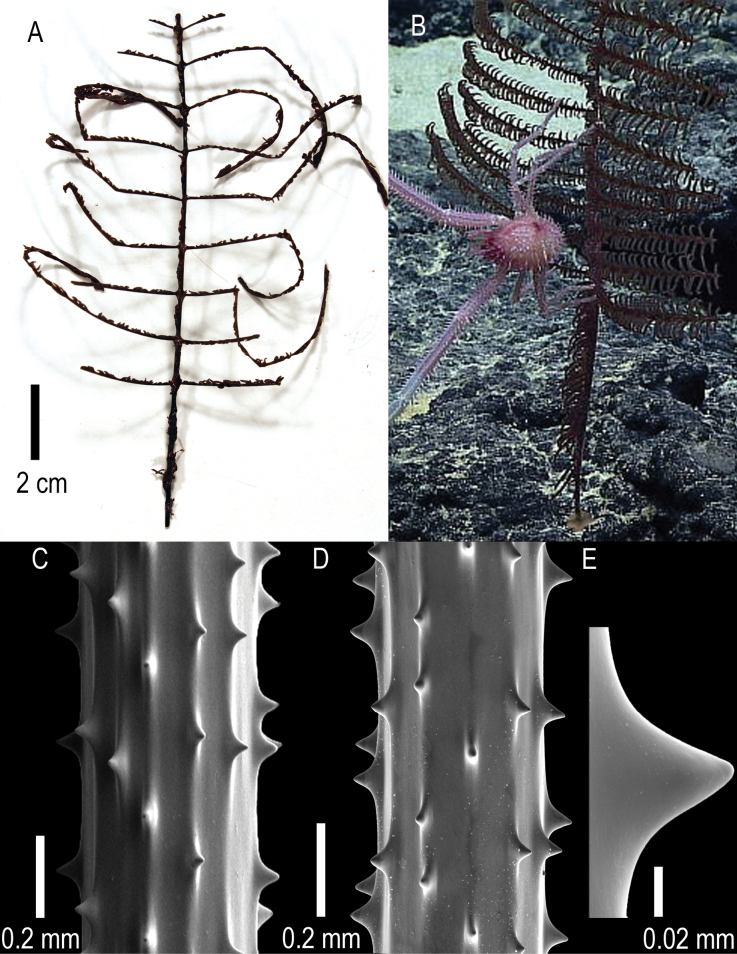
*Stauropathesmonopinnata* sp. nov. paratype (USNM 1424060) **A** collected colony **B** in situ image of colony **C, D** sections of pinnules showing skeletal spines **E** zoomed-in view of a singular skeletal spine.

**Figure 6.**
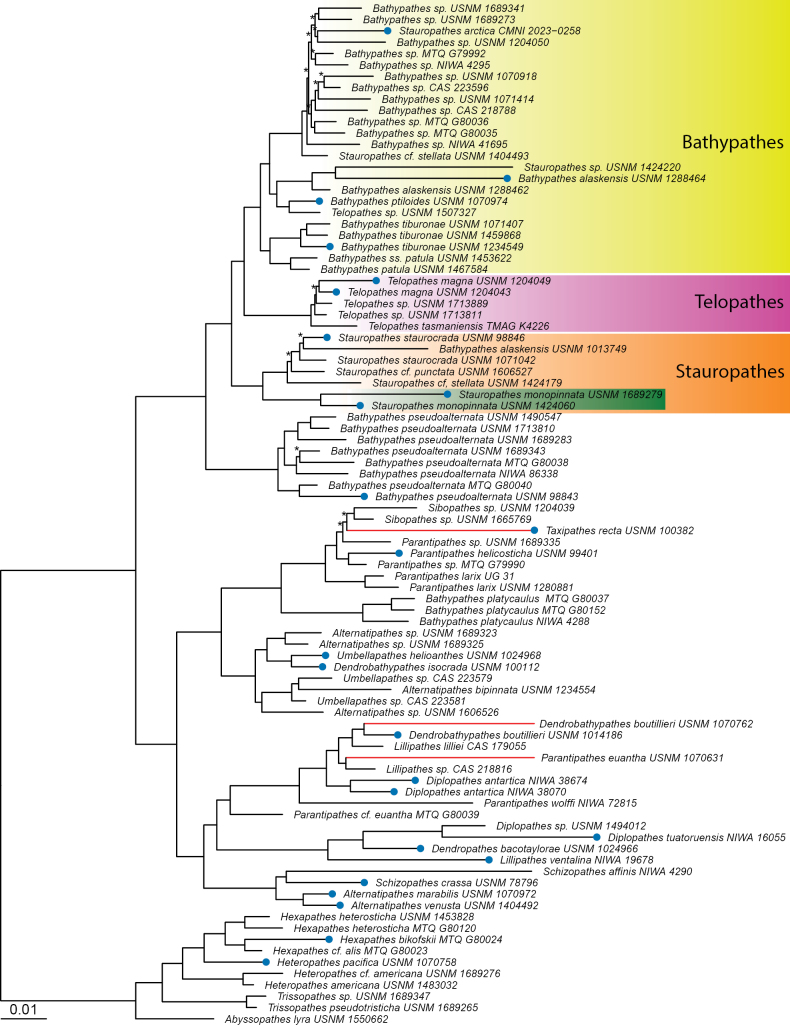
Maximum likelihood phylogeny of antipatharians of the families Schizopathidae and Cladopathidae based on a 50% complete matrix containing 794 loci. Taxa in green rectangle represent the holotype and paratype specimens of the new species. Ultrafast Bootstrap support values are 100% at all nodes unless noted with an asterisk (*). Red branches were artificially shortened and do not represent true branch length. The phylogeny rooted to Cladopathidae.

Figs 1 , 3 , 4 , 5 , 6 , Table 1 , Suppl. material 1

##### Material examined.

***Holotype*** • USNM 1689279, Whiting Bank, Puerto Rico, 17.8398°N, 65.6976°W, 738 m depth. Schmidt Ocean Institute R/V *Falkor (too)*, FKt230417, Health diagnostic of deep-sea coral, ROV *SuBastian* dive 518, April 29, 2023 (SEM stub No. 538). ***Paratype*** • USNM 1424060, 250 km southeast of Midway Atoll, Hawaii, 26.65°N, 175.4°W, 1604 m depth. NOAA (National Oceanic and Atmospheric Administration) R/V *Okeanos Explorer*, Cruise EX1603, Hohonu Moana: Exploring Deep Waters off Hawaii, ROV *Deep Discoverer* dive 5, March 5, 2016 (SEM stub 539).

##### Type locality.

Whiting Bank, Puerto Rico, 738 m depth.

##### Diagnosis.

Corallum monopodial, planar, unbranched, and pinnulate to the first order. Pinnules in two anterolateral rows and arranged in subopposite (almost opposite) pairs. Lowermost pinnules on stem 8 cm or more in length, decreasing in length towards apex of colony. Spines smooth and triangular with a rounded apex, 0.05–0.08 mm tall. Polyps 4–9 mm in transverse diameter, 2–3 mm interpolypar space, four to eight polyps counted per 5 cm.

##### Description of holotype.

The holotype (USNM 1689279) is a 15 cm tall monopodial colony (Fig. 3A–C). The unpinnulated section is about 2 cm based on in situ imagery because the basal plate and lowermost section of unpinnulated stem were not collected (Fig. 3C). The pinnulated section is 13 cm in length. Pinnules are simple, arranged in two anterolateral rows and in subopposite pairs (Fig. 3A, B). The pinnules are curved towards the abpolypar side of the colony. The lowermost pinnules on the stem are 8.3 cm in length, pinnules midway up the pinnulated section of the stem are 8 cm in length, and the uppermost pinnules on the stem are 4.2 cm in length. Diameter of the lowermost pinnule near the base of the pinnule is ~1 mm. Distance between pinnules in one row ranges from 1.3 to 1.7 cm and distance between pinnules of the same pair (i.e., on opposite sides of the stem) is < 1 mm, in some cases nearly opposite (Fig. 3B). Eight pinnules can be counted per 5 cm including pinnules in both rows. The distal angle of pinnules is 80–90° except for the uppermost pinnules, which are about 45° (Fig. 3A–C), and the interior angle formed by the subopposite pinnules is 160–180° (Fig. 3A).

Polyps are arranged in a single row (Fig. 3C, D). Polyps are 8–9 mm in the transverse diameter with 0.2–0.3 mm interpolypar space, resulting in polypar density ranging from 4 to 5 per 5 cm (Fig. 3E). Based on in situ imagery, tentacles exceed 1 cm when fully extended and the tissue color is dark red, and when preserved is dark red to dark brown.

Spines (Fig. 4A–C) are smooth and triangular with a rounded apex (Fig. 4C) that is at right angles to the axis or slightly inclined distally, with long and sloping distal and proximal edges. Polypar and abpolypar spines are 0.03 to 0.05 mm tall. The spines on pinnules are arranged in longitudinal rows, six to seven of which can be seen in one view (Fig. 4A, B). Spines are spaced 0.12–0.33 mm apart in a row, with about four to five spines per mm (Fig. 4A, B).

##### Description of the paratype.

The paratype (USNM 1424060) is a 14 cm tall colony (lowermost section of stem not collected) and the pinnulated section of stem is 12 cm (Fig, 5A). Based on in situ imagery (https://data.oceannetworks.ca/SeaTube?resourceTypeId=1000&resourceId=23621&diveId=3000&time=2016-03-06T00:56:55.000Z), the whole colony was approximated to be ~25 cm in length and the unpinnulated section of stem was ~7 cm in length. Striatum is present and distinct from the lower broken-off end of the stem to the lowermost pair of pinnules. Pinnules are simple, arranged in anterolateral rows and in subopposite pairs (Fig. 5A). The lowermost pinnules are 2.5 and 3.0 cm long; however, based on in situ imagery (Fig. 5B) the lowermost pinnules were the longest on the colony prior to subsampling. Pinnules midway up the pinnulated section of the stem are 8.0 cm long and the most distal pinnules are 1.3 cm long (Fig. 5A). Diameter near the base of the pinnule is ~1 mm. Distance between pinnules in one row ranges from 1.2 to 1.4 cm and distance between members of the subopposite pairs is < 1 mm (Fig. 5A). In some cases, the members of a pair appear to be directly opposite to one another. Ten pinnules can be counted per 5 cm including pinnules in both rows (Fig. 5A). Distal angle of pinnules is 80–90° and the interior angle formed between pinnules of a subopposite pair is 160–180° (Fig. 5A).

Polyps are arranged in a single row and are 4–6 mm in transverse diameter with an interpolypar space of 2 mm. Polypar density ranges from seven to eight per 5 cm. Based on in situ imagery, the tissue is dark red, and when preserved, it is brown to dark brown (Fig. 5B).

Spines are smooth, laterally compressed, and triangular with a rounded apex (Fig. 5C–E). Spines are 0.03–0.06 mm tall. The spines on pinnules are arranged in longitudinal rows, seven to eight of which can be seen in one view (Fig. 5C, D). Spines are spaced 0.23–0.36 mm apart in a row with about three to four spines per mm (Fig. 5C, D).

##### Intraspecies variation.

Both specimens possess simple pinnules with wide distal angles, arranged in slightly anterolateral rows, and in subopposite pairs that are almost opposite. Spacing between pinnules in one row is wide in both specimens: ≤ 1.7 cm in the holotype and ≤ 1.4 cm in the paratype, resulting in eight and ten pinnules per 5 cm. The spines on both specimens are short (≤ 0.06 mm tall), smooth, triangular with a rounded apex, and arranged in longitudinal rows, seven to eight of which are visible in lateral view. Tissue color is similar for both specimens; dark red in situ and a dark red-to-brown when preserved.

A minor difference between the holotype and paratype is that based on in situ footage, the paratype pinnules are more rigid than the holotype. A major difference between the holotype and paratype is their polyp characteristics, where the holotype has polyps 8–9 mm in transverse diameter with 0.2–0.3 mm interpolypar space, resulting in a density of four to five polyps per 5 cm, while the paratype’s smaller polyps are 4–6 mm in transverse diameter with 0.2 cm interpolypar space, resulting in a higher density of seven to eight per 5 cm. This is a notable difference that could be interpreted as the holotype and paratype representing different species. However, without additional specimens possessing similar morphological traits as these specimens with differing-sized polyps, it is premature to describe these two specimens as polyp size could vary within the species.

##### Phylogenetic results.

A total of 63–1052 conserved element loci were obtained per specimen. Total number of contigs ranged from 234,560 to 439,048,041 base pairs (bp) (average lengths ranged from 109 to 953 bp). The 50% taxon-occupancy matrix included 794 loci that were concatenated into an alignment with a total length of 385,232 bp. A 75% taxon-occupancy matrix, including 465 loci, was also run to compare results, and the topology in the region of the trees, including the new species, did not change. Read and locus summary statistics are detailed in Suppl. material 1.

The two specimens representing the new species with 100% branch support fell sister to the clade containing *Stauropathesstaurocrada* (USNM 98846 species- and genus-level holotype specimen, and USNM 1071042) (Fig. 5), supporting our decision to place the new species in *Stauropathes*. This clade also contains Stauropathescf.punctata (Roule, 1905) (USNM 1606527), Stauropathescf.stellata Opresko, 2019 (USNM 1424179), and *Bathypathesalaskensis* (USNM 1013749).

##### Comparative diagnosis.

*Stauropathesmonopinnata* sp. nov. differs from the four other species in the genus morphologically. The most prominent difference is that the new species is unbranched whereas the other species are branched. The new species also has wider distances between pinnules in a row; reaching 1.7 cm in the holotype compared to a maximum of 1.2 cm in *S.staurocrada* and *S.arctica* (Lütken, 1871), and 0.8 cm in *S.stellata* and *S.punctata*. Four of the species in the genus possess small spine heights less than 0.07 mm (spine measurements were not reported for the type of *S.punctata*). The species however, differ in the number of rows of spines that can be seen in one lateral view of a pinnule. The number of rows for the new species (six to eight visible on a pinnule diameter of 0.25 mm) is greater than that for *S.staurocrada* (four to six on pinnule diameter of 0.28 mm) and *S.stellata* (three to four on pinnule diameter of 0.28 mm) and less than *S.arctica* (nine to ten on pinnule diameter of 0.34 mm). The new species also has polyps that are 4 to 9 mm in transverse diameter, equal to or larger than *S.staurocrada* (4 mm), and the range includes polyps of *S.stellata* (6 mm) and *S.arctica* (7 mm). The transverse diameter of the polyps was not reported for *S.punctata*. A complete comparison of the morphological features of *Stauropathes* species can be found in Table 1.

**Table 1.** Comparison of species in the genus *Stauropathes*.

Feature	*S.monopinnata* sp. nov.	*S.monopinnata* sp. nov.	*S.staurocrada* ^c^	*S.stellata* ^d^	*S.punctata* ^e^	*S.arctica* ^f^
	holotype^a^	paratype^b^				
Corallum	unbranched	unbranched	branched	branched	branched	branched
Stem length (cm) (pinnulated / unpinnulated)	13 / 2	12 / 2 (unpinnulated section incomplete)	13 / not collected	6.3 / 6.1	21 / not collected	20 / 5
Pinnule diameter near base (mm)	1	1	1	0.5	1	Not reported
Pinnular angle (distal / interior)	45–90°/ 160–180°	80–90°/ 160–180°	60–70°/ 90–150°	80–90°/ 160–180°	80–90°/ 160–180°	80–90°/ 160–180°
Distance between pinnules on one side (mm)	13–17	12–14	8–12	5–10	6–8	12
Max Pinnule length (cm):	8.3	8	2	5.5	1	3
Pinnule density per 5 cm (both rows)	8	10	5–6	8	Not reported	Not reported
Number of orders of pinnules	1	1	1	1	1	>1?^g^
Spine height (mm)	0.03–0.05	0.03–0.06	0.04–0.06	0.06–0.07	Not reported	0.02–0.06
Spine ornamentation	Smooth	Smooth	Smooth	Smooth	Not reported	Smooth
Number of spine rows per view	6–7	7–8	4–6	3–4	Not reported	9–10
Space between spines in one row (mm)	0.12–0.17	0.11–0.44	0.12–0.24	0.21–0.33	Not reported	0.06–0.12
Spine density per 1 mm	4–5	4	8	4–6	Not reported	5
Polyp transverse diameter (mm)	8–9	4–6	2–4	5–6	Not reported	3–7
Polyp density per 5 cm	4–5	7–8	10–11	6–7	Not reported	Not reported
Number of polyps between adjacent pinnules in the same row	2	2	2	Not reported	Not reported	Not reported
Striatum	Lowest section not collected	Striations present until first row of pinnules	Present	Present from 2 cm above basal plate and extends 4 cm	Not reported	Not reported

^a^*Stauropathesmonopinnata* sp. nov. holotype (USNM 1689279) herein described. ^b^*Stauropathesmonopinnata* sp. nov. paratype (USNM 1424060) herein described. ^c^*Stauropathesstaurocrada* holotype (USNM 98846) in Opresko (2002). ^d^*Stauropathesstellata* holotype (USNM 16059) in Opresko (2019). ^e^*Stauropathespunctata* type series described in Roule (1905). ^f^*Stauropathesarctica*Brook’s (1889) translation of species described by Lütken (1871). ^g^ Unclear if ramification orders are pinnulated branches or pinnules.

While the lack of branches and the two rows of subopposite pinnules in the new species is typical of *Bathypathes* species, it differs morphologically in several ways: the distances between the members of each subopposite pair are smaller, and in some cases, the pinnules are almost opposite; the colonies have wider spaces between pinnules in a row, and the longest pinnules are also the lowermost ones on the stem.

##### Etymology.

The specific name derives from the Latin “mono” (one) and “pinnata” (feathered) referring to the new species general appearance due to the distinctive lack of branches compared to the other species in the genus.

##### Distribution.

Known from North Central Atlantic Ocean to North Pacific Ocean from 738 to 1604 m depth.

##### Discussion and conclusions.

This study presents morphological and molecular evidence to support the description of a new species within the genus *Stauropathes*. Furthermore, this study provides the most speciose molecular phylogeny of the family Schizopathidae to date, including specimens representing all 13 accepted schizopathid genera, holotypes or paratypes of 20 species, five of which also represent types at the genus level, and 12 species sequenced for the first time.

The new *Stauropathes* species, which lacks branches (like *Bathypathes*), required an emendation of the diagnosis of *Stauropathes* to include unbranched morphotypes. Additionally, the finding of one Bathypathes species in the *Stauropathes* clade and two *Stauropathes* species in the *Bathypathes* clade suggests they have a complicated evolutionary history, possibly driven by convergent evolution or hybridization.

Speciation is complex, and the phylogenetic models used in black coral studies have relied on maximum likelihood analyses with General Time Reversible substitution model, which does not account for processes like hybridization, recombination, or site-specific variation in substitution rates (Steenwyk et al. 2023). When dealing with complicated evolutionary histories, which seems to be the case for genera in Schizopathidae (see also ‘The Trigeneric Complex’ described in Bledsoe-Becerra et al. (2022)) a multispecies coalescent model should be used for estimating phylogenies while accounting for unresolved lineage sorting (Ramírez-Portilla and Quattrini 2023). This is an essential next step for resolving relationships at the species level for black corals.

## Supplementary Material

XML Treatment for Stauropathes

XML Treatment for Stauropathes monopinnata

## ﻿Additional information

### Author ORCIDs

Jeremy Horowitz https://orcid.org/0000-0002-2643-5200

Luke J. McCartin https://orcid.org/0000-0001-5374-3148

Samuel A. Vohsen https://orcid.org/0000-0003-1710-292X

Santiago Herrera https://orcid.org/0000-0001-7204-9434

### Data availability

Raw sequence reads were submitted to GenBank under BioProject # PRJNA1078781. Bioproject and biosample information for all specimens are listed in Suppl. material 1.

## Supplementary materials

Supplementary material 1 Read and locus summary statistics for all specimens included in the study Data type xlsx https://binary.pensoft.net/file/1279828This dataset is made available under the Open Database License (http://opendatacommons.org/licenses/odbl/1.0/). The Open Database License (ODbL) is a license agreement intended to allow users to freely share, modify, and use this Dataset while maintaining this same freedom for others, provided that the original source and author(s) are credited.Jeremy Horowitz, Mina Barajas, Luke J. McCartin, Samuel A. Vohsen, Santiago Herrera
